# Using mHealth Technology in a Self-Management Intervention to Promote Physical Activity Among Adults With Chronic Disabling Conditions: Randomized Controlled Trial

**DOI:** 10.2196/mhealth.6394

**Published:** 2017-12-01

**Authors:** Matthew Plow, Meghan Golding

**Affiliations:** ^1^ Frances Payne Bolton School of Nursing Case Western Reserve University Cleveland, OH United States

**Keywords:** disabled persons, exercise, self-care, mobile applications, motor activity, behavior, self-efficacy, goals, social support Introduction

## Abstract

**Background:**

Physical activity is considered a comprehensive approach for managing limitations in physical function among adults with chronic disabling conditions. However, adults with chronic disabling conditions often face many barriers to engaging in physical activity. A strategy to promote physical activity among adults with chronic disabling conditions is to encourage the use of mobile health (mHealth) apps.

**Objective:**

The objective of this pilot study was to examine the potential benefits of using commercially available mHealth apps in a self-management intervention among 46 adults with musculoskeletal or neurological conditions.

**Methods:**

Participants were randomized to one of 3 intervention groups: (1) mHealth-based self-management intervention, (2) paper-based self-management intervention, and (3) contact-control intervention. Participants in all 3 groups met in person once and received 3 follow-up phone calls with a trained graduate assistant. Participants in the mHealth-based and paper-based groups received a computer tablet or a paper diary, respectively, to facilitate goal setting, self-monitoring, and action planning. Participants in the contact-control group received information on healthy behaviors without being taught skills to change behaviors. The following outcomes were measured at baseline and at the 7th week: physical activity (Physical Activity and Disability Survey–revised), psychosocial factors (self-efficacy, self-regulation, and social support), and physical function (Patient Report Outcomes Measurement Information System, 6-min walk test, 1-min chair stands, and 1-min arm curls).

**Results:**

Repeated-measures multivariate analysis of variance (MANOVA) indicated significant differences between groups in physical activity levels (Wilks λ=0.71, *F*_6,76_=2.34, *P*=.04). Both the mHealth-based and paper-based groups had large effect size increases in planned exercise and leisure-time physical activity compared with the contact-control group (Cohen *d*=1.20 and *d*=0.82, respectively). Repeated-measures MANOVA indicated nonsignificant differences between groups in psychosocial factors (Wilks λ=0.85, *F*_6,76_=1.10, *P*=.37). However, both the mHealth-based and paper-based groups had moderate effect size improvements in self-efficacy (*d*=0.48 and *d*=0.75, respectively) and self-regulation (*d*=0.59 and *d*=0.43, respectively) compared with the contact-control group. Repeated-measures MANOVA indicated nonsignificant differences between groups in physical function (Wilks λ=0.94, *F*_8,66_=0.27, *P*=.97). There were small and nonsignificant changes between the mHealth-based and paper-based groups with regard to most outcomes. However, the mHealth-based group had moderate effect size increases (*d*=0.47) in planned exercise and leisure-time physical activity compared with the paper-based group.

**Conclusions:**

We found that using commercially available mHealth apps in a self-management intervention shows promise in promoting physical activity among adults with musculoskeletal and neurological conditions. Further research is needed to identify the best ways of using commercially available mobile apps in self-management interventions.

**Trial Registration:**

Clinicaltrials.gov NCT02833311; https://clinicaltrials.gov/ct2/show/NCT02833311 (Archived by WebCite at http://www.webcitation.org/6vDVSAw1w)

## Introduction

### Background

Promoting engagement in physical activity is an important strategy for reducing the consequences of musculoskeletal and neurological conditions [1]. For example, engaging in physical activity can help mitigate limitations in physical function, which is a hallmark consequence of several musculoskeletal and neurological conditions, such as osteoarthritis, fibromyalgia, systemic lupus erythematosus, stroke, Parkinson disease, and multiple sclerosis [2,3]. Common symptoms of these conditions, such as fatigue, pain, and muscle weakness, can result in physical limitations such as difficulty walking and accomplishing daily chores. Engaging in physical activity can reduce the impact of these common symptoms and help prevent limitations in physical function [2-4]. However, adults with musculoskeletal and neurological conditions are largely sedentary [5,6] because they often encounter barriers that reduce their ability and motivation to engage in physical activity [7-9].

A possible solution for promoting physical activity in adults with musculoskeletal and neurological conditions is delivering self-management interventions [10]. Self-management interventions can encourage the learning of skills (eg, goal setting, communication, and self-regulation) that improve psychosocial factors (eg, self-efficacy and social support) and promote engagement in physical activity [11]. Self-management interventions are effective in a variety of delivery formats, including in-person and remote formats (eg, phone, print, and Internet) [11,12]. Self-management interventions delivered remotely may be as effective as those delivered in person [13,14]. Furthermore, using mobile health applications (mHealth apps) in self-management interventions may help promote physical activity [15]. However, few studies have systematically evaluated whether there are any added benefits of using mHealth apps in self-management interventions among adults with musculoskeletal and neurological conditions.

Incorporating mHealth apps into self-management interventions may have several advantages among adults with musculoskeletal and neurological conditions. [16]. mHealth apps can be used to self-monitor symptoms, set goals, and learn self-management skills. Importantly, mHealth apps may help facilitate the tailoring of self-management interventions and can provide feedback, reminders, and information that can be tailored to encourage physical activity. For example, mHealth apps can provide immediate feedback on physical activity goals and health status via graphs and short messages; remind participants to engage in physical activity; and help tailor content to accommodate preferences for information, aesthetics, and learning style. These functionalities may increase perceived relevance, thereby, making it more likely for participants to think about and act upon recommendations [17]. Studies are needed to confirm whether these potential advantages of using mHealth apps in self-management interventions translate into better outcomes among adults with musculoskeletal or neurological conditions.

Although there are numerous self-management studies of mHealth apps in healthy populations and in adults with heart disease, cancer, and diabetes, there are far fewer studies among adults with musculoskeletal or neurological conditions [16,18-20]. Furthermore, most self-management research on mHealth apps has focused on developing and testing an app for a single chronic condition [16,21], so generalizability of mHealth apps in promoting healthy behaviors across the population with musculoskeletal and neurological conditions remains unknown. Existing research indicates that mHealth apps may be effective in promoting healthy behaviors in healthy populations and in adults with heart disease, cancer, and diabetes [16,18-20]. However, questions remain about the feasibility and benefits of using mHealth apps in self-management interventions among adults with musculoskeletal or neurological conditions. Studies examining the benefits of using commercially available mHealth apps in self-management interventions may help inform clinical recommendations and prioritize research. Clinicians will be able to make informed decisions about using commercially available mHealth apps to encourage the self-management of symptoms. Furthermore, researchers will be able to make informed decisions about the merits of developing new apps versus refining existing apps for people with disabling conditions.

### Objectives

We conducted a randomized controlled pilot study to examine the potential benefits of a self-management intervention that was augmented with mHealth apps used on a computer tablet among 46 adults with musculoskeletal or neurological conditions. Recruitment was focused on including adults who have musculoskeletal or neurological conditions that characteristically result in physical limitations, such as osteoarthritis, fibromyalgia, systemic lupus erythematosus, stroke, Parkinson disease, and multiple sclerosis. Participants were randomized into one of 3 groups: (1) self-management intervention augmented with commercially available mHealth apps used on a computer tablet (ie, mHealth-based group), (2) self-management intervention augmented with paper diary (ie, paper-based group), and (3) information-only intervention (ie, contact-control group). We selected these 3 groups to examine the effects of the self-management interventions while controlling for the number of contacts with the interventionist. Here, we report on the primary outcome—physical activity—and the secondary outcomes related to physical activity—psychosocial factors and physical function. We tested the following hypotheses:

- In comparison with the contact-control group, both the mHealth-based and paper-based groups will yield significant increases in physical activity, with the mHealth-based group yielding a significantly larger increase.
- In comparison with the contact-control group, both the mHealth-based and paper-based groups will yield significant improvement in psychosocial factors (ie, self-efficacy, self-regulation, and social support), with the mHealth-based group yielding a significantly larger increase.
- In comparison with the contact-control group, both the mHealth-based and paper-based groups will yield significant increases in physical function, with the mHealth-based group yielding a significantly larger increase.

## Methods

### Overview

A randomly allocated, 3-group, single-blinded repeated-measures design was used to generate pilot data to test the hypotheses. Participants (n=46) were recruited via community outreach and randomly allocated to one of 3 groups using an allocation ratio of 1:1:1. Participants in all 3 groups were asked to attend 1 in-person session and partake in 3 follow-up phone calls over a 6-week period with a trained research assistant who had a bachelor’s degree in health education. Participants in the mHealth-based group and paper-based group were asked to track their progress in meeting self-management goals using a Google Nexus 7 tablet (ASUS, Taiwan) or paper diary, depending on their group assignment. Participants in the contact-control group received information on healthy behaviors. Regardless of the group assignment, all participants received the Google Nexus tablet at the completion of the study. A research assistant blinded to group assignment administered self-report questionnaires and a physical assessment at baseline and at 7th week. The Cleveland Clinic and University Hospitals Institutional Review Board approved this study.

### Participants and Procedures

We aimed to recruit 12 to 16 participants in each group, which is consistent with recommendations by Dobkins et al [22] on obtaining stable effect size estimates in pilot studies. Participants were recruited through physician referrals, postings in physician offices, visiting support groups, advertising in e-newsletters, and postings on Facebook and community bulletin boards. The study was advertised as a comparison of different health and wellness programs meant to examine the benefits of using mHealth apps on a computer tablet.

To help ensure adults with disabilities had opportunities to fully participate in the study, we followed universal design principles for research [23]. This included recruiting participants through a variety of media (ie, print and audio), allowing multiple options for responding to recruitment notices (ie, phone and Internet), using large print on recruitment flyers, informed consent, and intervention handouts, and incorporating multiple methods for responding to questionnaires (ie, both audio and visual).

Inclusion criteria were: physician-confirmed diagnosis of a disease of the nerves, muscles, or bones that characteristically results in physical limitations, physician’s consent to engage in a physical activity program, age between 18 to 76 years, engagement in 90 min or less of purposeful physical activity each week, engagement in unhealthy eating habits (ie, <10 on a questionnaire about nutritional habits) [24], score ≤17 on mental composite and ≤16 on physical composite of the Global Health Questionnaire [25], and access to Internet at home or at a library or a community center. The study criteria for age were changed during the study to include the oldest adults in the study that met all other study criteria. The Global Health Questionnaire [25] was selected from the Patient Reported Outcomes Measurement Information System (PROMIS) [26]. The Global Health Questionnaire generates a mental and physical health status composite score. The 2 composite scores can be compared with those of the general population. We used this measure to help reduce ceiling effects by selecting cutoff scores that would exclude participants who were healthier than the general population in mental and physical health status. Thus, participants in the study may or may not have had a disability, but had a definite diagnosis of a musculoskeletal or neurological condition. For example, a participant could have a diagnosis of multiple sclerosis, but not have a disability or impairment that would limit participation in daily activities and social roles. Exclusion criteria were: report >3 falls per month, comorbid conditions that significantly limit engagement in physical activity (eg, chronic heart failure, myocardial infarction, or uncontrolled diabetes mellitus), severe cognitive deficits (ie, a weighted score of less than 12 on the short version of the Blessed Orientation Memory Concentration test) [27], or report of existing use of mHealth apps or a paper diary to track behaviors.

### Randomization and Blinding

Once consent and baseline data were collected, participants were randomly allocated using a numbered series of 48 prefilled envelopes in blocks of 3, using a random number generator. A research assistant not involved in interacting with the participants put the group assignment in an envelope and sealed it. The research assistant who delivered the first in-person session was responsible for opening the randomization envelope. Research assistants who were responsible for administering the questionnaires and physical assessments were blinded to group assignment. It was not feasible to blind research participants to group assignment.

### mHealth-Based and Paper-Based Self-Management Interventions

Participants randomized to the mHealth-based group or the paper-based group received the same number of contacts (ie, 1 in-person session plus 3 phone calls) and behavior change techniques. Social cognitive theory inspired both self-management interventions [28,29]. We implemented strategies to enhance self-efficacy (ie, mastery, persuasion, modeling, and appraisal), increase self-regulation subfunctions (ie, setting, monitoring, and achieving goals), and decrease the perceived barriers to the regulation of motivation. We used the behavior change techniques of instruction, goal setting, self-monitoring, action planning, social support, information on the benefits and consequences of behaviors, and barrier management as defined by Michie et al [30]. Participants were taught how to track progress in meeting physical activity goals and overcome barriers to engaging in a physical activity program. Goal attainment scaling helped participants develop detailed intentions and define success for engaging in a physical activity program. Participants were asked to set goals and develop action plans related to physical activity and nutrition. Details about the nutritional goals and related outcomes are reported elsewhere.

#### Physical Activity Program

During the in-person session, a personalized physical activity program was developed using an interview guide administered by the research assistant. The first author developed and refined the interview guide in previous studies [31,32]. Participants were asked about their physical activity habits, what they enjoyed and disliked about physical activity, and the barriers encountered to engaging in physical activity. Participants were asked whether they preferred incorporating physical activity into daily routine or setting aside specific times to engage in an exercise program (eg, one 30-min bout or ten 3-min bouts of physical activity). On the basis of participants’ responses, recommendations were made either to engage in a physical activity program using a pedometer or to set aside time for a home exercise program. The pedometer-based program consisted of learning strategies to increase step counts throughout the course of the day. The home exercise program consisted of stretching and cardiovascular, strength, and balance training performed using a chair and resistance bands. The program was personalized to the participant’s fitness level. Participants were asked to engage in the home exercise program for 30 min, 3 to 5 days a week.

#### Phone Calls

Participants received 3 follow-up phone calls at a frequency of 1 call placed every other week for 6 weeks after the in-person session. The phone calls were delivered by the same research assistant who delivered the in-person session. During each phone call, adverse events were monitored, questions about engaging in the physical activity program were answered, and education was provided regarding overcoming barriers. The first phone call focused on reinforcing the benefits of engaging in physical activity and setting goals for engaging in physical activity. The second phone call focused on fostering social support for engaging in physical activity. The third phone call focused on managing specific symptoms that were barriers to engaging in the physical activity program. Recommendations were made to continue the physical activity program without changes, modify the types of exercises being performed, or increase the frequency or intensity of exercise. At the end of each phone call, participants were reminded to use the mobile apps or paper diary.

#### mHealth-Based Self-Management Intervention Group

Participants received a Google Nexus tablet (ASUS, Taiwan) and were shown how to use it during the first in-person session. We decided to provide participants with a tablet rather than a mobile phone because the screen is larger and may be more user-friendly for adults who experience mobility or sensory impairments. Participants learned how to use the following mobile apps downloaded at the Google Play Store: Lose it! (FitNow, Inc, Boston, MA, USA), iPro Habit Tracker Free (IntelliPro, Atlantis Enclave, India), and Memories: The Diary (Victor Nakonechny, Heidelberg, Germany). We selected these apps because of their popularity, positive reviews, relevance in augmenting self-management interventions, and flexibility in customizing goals and self-monitoring progress, and because they were free to download and use. Participants used the Lose it! app to track physical activity and nutrition behaviors; the iPro Habit Tracker app to track progress in achieving goals; and the Memories app to monitor and describe problematic symptoms. The research assistant asked participants to use the apps at least once a day and review progress in meeting goals at least once a week using the graph or feedback functionalities available with these apps. Participants were shown how to modify settings in each of the apps to accommodate preference about information, aesthetics, goal setting, and self-monitoring. The research assistant asked participants to demonstrate use of each of the apps to ensure proficiency and address potential usability barriers. Any additional questions or difficulties in using the apps were addressed during the phone calls by the same research assistant. No major revisions or updates occurred to the apps during the study.

#### Paper-Based Self-Management Intervention Group

Participants received a paper diary to track physical activity and nutrition behavior, monitor progress in meeting goals, and describe problematic symptoms. During the in-person session, participants’ goals were written in the diary, and they were instructed on how to track their behaviors and monitor their symptoms. The diary had been refined in previous studies to improve efficiency in self-monitoring behavior. Tracking of behaviors involved circling icons or numbers corresponding to participants’ goals. They were asked to track their behavior each day, review their journal weekly, and tally the numbers to examine whether they were on track to meet their goals.

### Contact-Control Intervention Group

Participants randomized to the contact-control group received the same number of contacts as participants in the mHealth-based and paper-based groups. During the first in-person session, participants received generic education on physical activity and nutrition guidelines for people with disabilities. At each subsequent call, we monitored for adverse events and asked about their overall health and well-being. Phone calls were placed at the same frequency with a similar length of time as phone calls placed in the mHealth-based and paper-based groups. Participants were provided additional information about different forms of physical activity one could engage in and suggestions about making healthy food choices, but goals were not set and skills to change behavior (ie, goal setting, self-monitoring, and barrier management) were not taught. To encourage retention in this group, participants had the option to receive the self-management intervention at the completion of the study.

### Treatment Fidelity

We followed guidelines established by Bellg et al [33] for measuring and maintaining treatment fidelity. Manuals of operating procedures were provided to research assistants and incorporated into their training. Standardized training was provided to research assistants who delivered the intervention and administered the outcome measures. Training included 15 hours of readings and tutorials on understanding how to support self-management of symptoms, encourage behavior change, and conduct clinical trials. Adherence to the intervention protocol was monitored using checklists completed by research assistants and episodic monitoring of the sessions by the first author. Feedback on protocol adherence and ongoing training occurred in follow-up meetings as needed. Fidelity of interventions was further monitored by incorporating process measures, reviewing entries in mHealth apps or paper dairy, and using goal attainment scaling (described below).

### Measurements

#### Physical Activity

We used the Physical Activity and Disability Survey–revised (PADS-revised) to comprehensively measure physical activity behavior [34,35]. We selected this survey because it is designed to be relevant across different disabling conditions and can measure the frequency and intensity of different types of physical activity to generate continuous composite scores. Rimmer et al originally developed the PADS. They found the PADS to have significant correlations with absolute peak volume oxygen (VO_2_), relative peak VO_2_, maximum workload, and time to exhaustion [36]. In 2 subsequent studies, Kayes et al revised the PADS to facilitate understanding of questions and improve the methods of scoring the scale. PADS-revised comprises 5 subscales: planned exercise/leisure-time physical activity, general physical activity, therapy, employment, and wheelchair use [34,35]. The revised scale has adequate test-retest reliability and distinct subscales. We used the scoring algorithm provided by Kayes et al to calculate the subscales. A higher score indicates a greater amount of physical activity. For the analyses, we used 3 subscales: planned exercise/leisure-time physical activity, general physical activity, and employment physical activity. In our sample, the PADs-revised total composite score at baseline had concurrent validity with self-reported physical function (*r*=.54), the number of chair stands in 1 min (*r*=.37), and the 6-min walk test (*r*=.41).

#### Self-Efficacy

We used the Exercise Confidence Survey developed by Sallis et al [37]. The survey asks about confidence in sticking to an exercise program and making time for exercise. For example, “How sure are you that you can: stick to your exercise program after a long, tiring day at work; exercise even though you are feeling depressed; and stick to your exercise program when social obligations are very time-consuming?” Cronbach alpha in this study was .92. Thus, the 12 items were averaged to generate a single composite score. A higher score indicates increased confidence to engage in exercise.

#### Social Support

We used the Social Support and Exercise Survey developed by Sallis et al [38]. The survey asks about the amount of support that family and friends provide for engaging in exercise. For example, “How often family or friends: exercise with me; give encouragement to stick with my exercise; make positive comments about my physical appearance; and take over chores so I have more time to exercise?” Cronbach alpha for family and friends in this study was .92. Thus, the 13 items for family and friends were averaged to generate a single composite score. A higher score indicated increased social support from family and friends for regular exercise.

#### Self-Regulation

We used the Goal Setting for Exercise Scale developed by Rovniak et al [39]. Questions pertain to the frequency of setting exercise goals and how goals are monitored and achieved. For example, “I often set exercise goals; I usually set dates for achieving my exercise goals; and if I do not reach an exercise goal, I analyze what went wrong.” Cronbach alpha in this study was .91. Thus, the 10 items were averaged to generate a single composite score. A higher score indicates increased use of self-regulation strategies in setting and achieving exercise goals.

#### Self-Report Physical Function

We used the PROMIS for self-report physical function [26,40]. PROMIS was developed through an initiative by the National Institutes of Health. Participants completed the computer adaptive test version that assesses physical function or an individual’s ability to carry out activities that requires physical action. It includes questions on lower and upper extremity function. Participants are asked about their abilities to perform a wide range of tasks. A T-score is automatically calculated after the participant completes the questionnaire using the PROMIS Assessment Center website. A higher T-score indicates better physical function compared with the general population. The PROMIS physical function questionnaire has been found to be valid and reliable in adults with neurological and musculoskeletal conditions [26,40].

#### Performance-Based Physical Function

We administered adapted portions of the Senior Fitness Test [41], which consisted of the 6-min walk test, 1-min chair stands, and 1-min arm curls. A trained research assistant blinded to group assignment administered the assessment. The research assistant provided standardized instructions to each participant and was instructed to be consistent in timing and number of verbal cues delivered during the assessment. Participants were asked to walk as fast as they could or perform as many repetitions as possible, but they could take as many rest breaks as needed during the timed tasks.

#### Process Measures

Several process measures were incorporated to monitor treatment fidelity and examine the feasibility of conducting a larger clinical trial. Process measures included attrition rate, adverse events, the percentage of phone calls completed following the manual, percentage of entries in the mHealth apps, and percentage of entries in the paper diary. The percentage of mHealth apps and paper diary entries was calculated by dividing the number of actual entries by the total number of days they were supposed to have entries. To further monitor treatment fidelity and help offset biases associated with using a diary to measure the achievement of a goal, we used goal attainment scaling (GAS) following the recommendations of Turner-Stokes et al [42,43].

GAS is a valid strategy for quantifying the achievement of each participant’s goal [42,43]. GAS can be used to examine the extent to which each participant’s personalized goals are met due to an intervention. Participants in the mHealth-based group and the paper-based group collaborated with the research assistant to set specific, measurable, achievable, relevant, and timed goals. The goal and its attainment were formulated to reflect the participants’ motivation and preference for engaging in physical activity and their desire to improve their physical function. Goals consisted of increasing the frequency, duration, or intensity of engaging in exercise or specifying improvements in daily physical activities. For example, common goals pertained to improving balance, increasing walking distances, and engaging in exercise 3 to 5 days per week. Attainment of the goal was quantified on a scale between −2 and +2 in which a response of 0 indicated achieving the goal at the expected level and a response of +2 or −2 indicated achieving the goal much more or much less than the expected level. The participants worked with the research assistant to define each level of attainment at the first visit. At the posttest visit, participants were asked to rate their achievement of the goal on the −2 to +2 scale. An unweighted T-score was then calculated.

### Analysis

We first determined whether the data met the assumption to conduct parametric statistical testing. We then conducted an efficacy analysis using a 3 (group: mHealth-based vs paper-based vs control) × 2 (time: baseline and 7 weeks) multivariate analysis of variance (MANOVA). In total, 3 repeated-measures MANOVAs were conducted to test hypotheses on (1) physical activity levels (ie, planned exercise/leisure-time physical activity, general physical activity, and employment physical activity), (2) psychosocial factors (ie, self-efficacy, self-regulation, and social support), and (3) physical function (ie, PROMIS physical function, 6-min walk test, 1-min chair stands, and 1-min arm curls). If the multivariate test was significant, interactions were examined using post hoc analyses without concern for family wise error. The univariate *F* test was used to examine which dependent variables were significantly different across all 3 groups. Paired and independent samples *t* tests were used to examine whether the dependent variable significantly changed across time and between groups. For variables that were not normally distributed, we also used Wilcoxon signed rank test and Kruskal–Wallis test. The analyses were conducted using IBM SPSS Statistics for Windows, version 24.0 (IBM Corp, Armonk, NY, USA).

We calculated effect sizes as standardized mean differences while adjusting for baseline differences and correlations between measures, and dividing by the pooled standard deviation [44]. A small effect was considered to be 0.20; a medium effect was considered to be 0.50; a large effect was 0.80 [45]. Independent samples *t* tests were used to compare the mHealth intervention with the paper diary intervention on the GAS and the percentage adherence in self-monitoring physical activity behaviors (ie, process measures).

## Results

Demographic characteristics, type and number of chronic conditions, and disability status for each group are presented in Tables 1 and 2. The average age of the sample was 57.8 years. Majority of the research sample comprised females (84%); at least 13% of the sample had a household income ≤US $25,000 and 17% of the sample was non-white. Participants had an average of 1.5 chronic conditions and 39% of the sample had multiple chronic conditions. The most common conditions were fibromyalgia, multiple sclerosis, osteoarthritis, and Sjögren syndrome. Average time since diagnosis was 13.3 years. Common comorbid conditions included hypothyroidism, diabetes, and high blood pressure. Common symptoms that were described by participants as interfering with engagement in physical activity were fatigue, pain, muscle weakness, and balance problems. On the basis of the screening questionnaire used to determine eligibility (ie, Global Health Questionnaire) [25], the sample was about half to one standard deviation below the general population in terms of global mental and physical health. On the basis of the PROMIS physical function scale at baseline [40], the sample was about one standard deviation below the general population.

**Figure 1:**
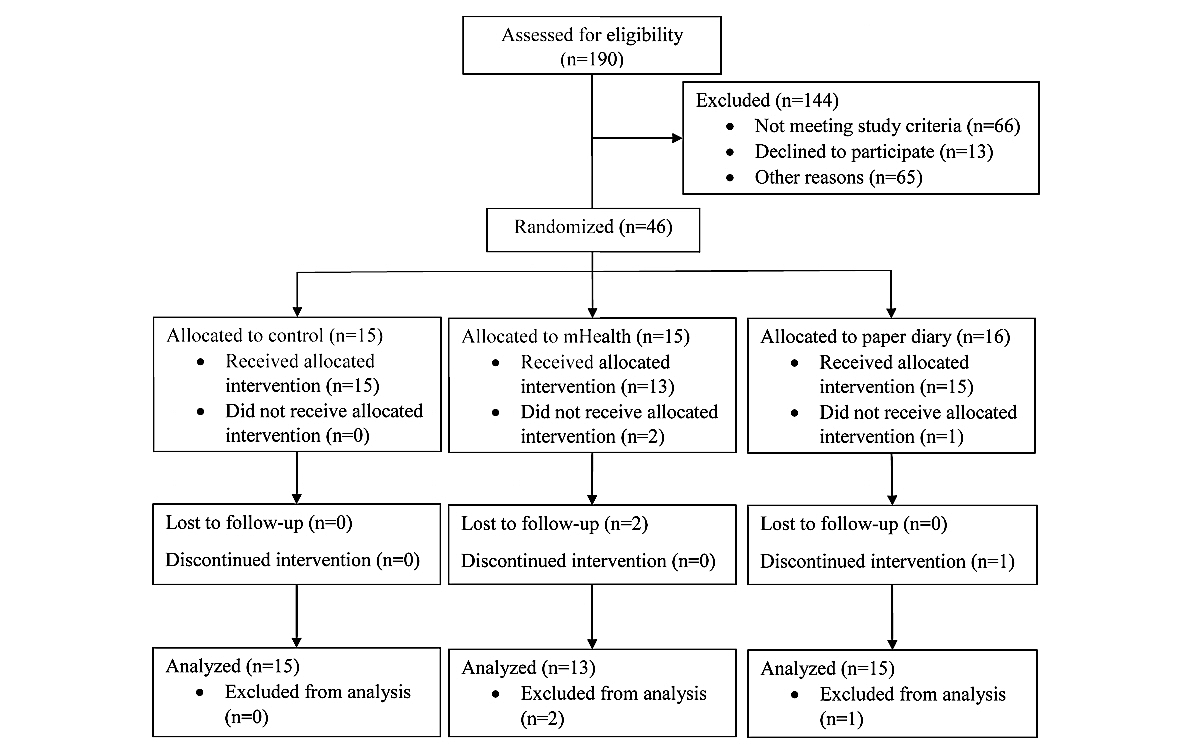
Flowchart.

Figure 1 illustrates the CONSORT flowchart. All dependent variables had Pearson correlations of <.6, indicating that multicollinearity was not a problem. Nonsignificant Box’s M tests for each of the MANOVAs indicated homogeneity of covariance matrices for the dependent variables across the 3 groups. Most dependent variables for each group were normally distributed based on skewness and kurtosis values and nonsignificant Shapiro–Wilk test of normality. However, the PADs-revised subscale of exercise/leisure-time physical activity was skewed. Thus, we report the results below using both MANOVAs and nonparametric statistics.

The attrition rate for the study was 6.5%. In total, 6 adverse events were possibly related to the study. The adverse events (ie, mHealth-based group=2, paper-based group=3, and contact-control group=1) included mild to moderate musculoskeletal injury resulting from a fall during engagement in physical activity or performance of daily chores.

**Table 1:** Characteristic of sample (mean and standard deviation).

Characteristics	Mean (SD^a^)
Age (years)	57.80 (9.48)
Global mental health scale	13.15 (1.94)
Global physical health scale	12.50 (1.94)
Body mass index	31.69 (8.82)

^a^SD: standard deviation.

**Table 2:** Characteristic of sample (frequency).

Characteristics		Frequency count, n (%)
Gender (female)		39 (85)
Number with multiple chronic conditions		18 (39)
**Type of conditions (7 most common)**		
	Fibromyalgia	17 (37)
	Multiple sclerosis	12 (26)
	Osteoarthritis	12 (26)
	Sjögren syndrome	11 (24)
	Parkinson disease	5 (11)
	Chronic fatigue syndrome	4 (9)
	Systemic lupus erythematosus	3 (7)
**Type of comorbid conditions (3 most common)**		
	High blood pressure	12 (26)
	Type II diabetes	5 (11)
	Hypothyroidism	5 (11)

A total of 87% of participants completed all 3 phone calls as intended in the mHealth-based group; 94% completed all 3 phone calls as intended in the paper-based group; and 93% completed all 3 phone calls as intended in the contact-control group. There were no significant differences between the mHealth-based and paper-based groups in terms of GAS. However, participants were significantly (*P*=.003) more likely to keep track of physical activity behaviors using a paper diary (84.3%) compared with a tablet computer (48.2%). Means, standard deviations, and effect sizes of the outcome measures are presented in Table 3.

Hypothesis 1: In comparison with the contact-control group, both the mHealth-based and paper-based groups will yield significant increases in physical activity, with the mHealth-based group yielding a significantly larger increase.

**Table 3:** Means, standard deviations, and effect sizes of outcome measures.

Measures		Pretest	Posttest	Paper versus control	mHealth versus control	mHealth versus paper
		Mean (SD^a^)	Mean (SD)	Effect size (Cohen *d*)	Effect size (Cohen *d*)	Effect size (Cohen *d*)
**PADs-revised^b^: total composite**				0.35	0.63	0.16
	mHealth^c^	0.36 (0.77)	0.76 (0.92)			
	Paper	0.31 (1.01)	0.55 (1.15)			
	Control	0.43 (0.68)	0.36 (0.61)			
**PADs-revised subscale: exercise/leisure-time physical activity**				0.82	1.20	0.47
	mHealth	−0.28 (0.98)	0.38 (1.04)			
	Paper	−0.09 (0.87)	0.14 (0.65)			
	Control	0.08 (0.79)	−0.30 (0.67)			
**PADs-revised subscale: general physical activity**				−0.25	−0.50	−0.22
	mHealth	0.72 (0.91)	0.78 (0.84)			
	Paper	0.60 (0.97)	0.89 (1.23)			
	Control	0.55 (1.16)	1.12 (1.04)			
**PADs-revised subscale: Employment physical activity**				0.31	0.43	0.04
	mHealth	0.22 (1.06)	0.35 (1.04)			
	Paper	0.20 (1.34)	0.28 (1.46)			
	Control	0.15 (0.98)	−0.13 (0.50)			
**PROMIS^d^: physical function**				−0.01	−0.08	−0.08
	mHealth	39.87 (5.87)	38.44 (7.54)			
	Paper	38.89 (3.14)	37.91 (4.52)			
	Control	42.44 (5.45)	41.51 (3.96)			
**6-min walk test (meters)**				−0.06	0.16	0.19
	mHealth	303.69 (87.23)	327.46 (68.32)			
	Paper	316.32 (117.58)	320.61 (122.02)			
	Control	362.32 (91.01)	385.23 (82.26)			
**Bicep curls (count, 1 min)**				−0.22	−0.29	−0.09
	mHealth	23.85 (10.04)	24.23 (11.97)			
	Paper	22.40 (8.68)	23.73 (8.86)			
	Control	24.13 (8.63)	27.40 (8.77)			
**Chair stands (count, 1 min)**				0.03	−0.02	−0.04
	mHealth	15.00 (6.66)	16.69 (8.38)			
	Paper	13.73 (6.64)	15.73 (7.93)			
	Control	16.47 (5.13)	18.27 (5.40)			
**Exercise confidence survey**				0.75	0.48	−0.17
	mHealth	3.26 (0.96)	3.25 (0.82)			
	Paper	3.29 (0.91)	3.42 (0.76)			
	Control	3.33 (0.45)	2.97 (0.55)			
**Goal setting for exercise scale (self-regulation)**				0.43	0.59	0.13
	mHealth	1.98 (0.83)	2.71 (0.96)			
	Paper	2.57 (0.96)	3.17 (0.98)			
	Control	1.99 (0.93)	2.19 (0.79)			
**Social support and exercise survey**				0.38	0.44	0.01
	mHealth	1.58 (0.34)	1.79 (0.56)			
	Paper	1.85 (0.63)	2.05 (0.75)			
	Control	2.13 (0.80)	2.03 (0.83)			

^a^SD: standard deviation.

^b^PADs-revised: Physical Activity and Disability Survey–revised.

^c^mHealth: mobile health.

^d^PROMIS: Patient Report Outcomes Measurement Information System.

The MANOVA to test hypothesis #1 indicated that the condition by time interaction was significant (Wilks λ=.71, *F*_6,76_=2.34, *P=*.04). The univariate *F* test indicated that planned exercise/leisure-time physical activity was significantly different across the 3 groups (*F*_2,40_=5.02, *P*=.01), which was consistent with the Kruskal–Wallis Test (χ^2^_2_=6.4, *P*=.04). Both parametric and nonparametric tests indicated that the mHealth-based group had a significant and large increase in the planned exercise/leisure-time physical activity subscale of the PADS-revised (*t*_12_=−3.03, *P*=.01; *Z*=−2.49, *P*=.01) over time, whereas the control group had a small and nonsignificant change in the planned exercise/leisure-time physical activity subscale of the PADS-revised (*t*_14_=1.38, *P*=.19; *Z*=−1.16, *P*=.25) over time. The paper-based group also had a nonsignificant change in the planned exercise/leisure-time physical activity (*t*_14_=−1.27, *P*=.23; *Z*=−.94, *P*=.35) over time. There were nonsignificant differences between the mHealth-based and paper-based groups in promoting physical activity. However, the mHealth-based group had a moderate effect size (*d*=0.47) in planned exercise and leisure-time physical activity compared with the paper-based group.

Hypothesis 2: In comparison with the contact-control group, both the mHealth-based and paper-based groups will yield significant improvement in psychosocial factors (ie, self-efficacy, self-regulation, and social support), with the mHealth-based group yielding a significantly larger increase.

The repeated-measures MANOVA to test hypothesis #2 indicated that the condition by time interaction was not significant (Wilks λ=.85, *F*_6,76_=1.10, *P*=.37). However, both the mHealth-based and paper-based groups had moderate effect sizes in self-efficacy (*d*=0.48 and *d*=0.75; respectively) and self-regulation (*d*=0.59 and *d*=0.43, respectively) compared with the contact-control group. Both the mHealth-based and paper-based groups had small effect sizes in social support compared with the contact-control group. There was a small and nonsignificant difference in psychosocial factors between the mHealth-based and paper-based groups.

Hypothesis 3: In comparison with the contact-control group, both the mHealth-based and paper-based groups will yield significant increases in physical function, with the mHealth-based group yielding a significantly larger increase.

The repeated-measures MANOVA to test hypothesis #3 indicated that the condition by time interaction was not significant (Wilks λ=.94, *F*_8,66_=0.27, *P*=.97). All 3 groups had small within- and between-subject effects in influencing physical function. The negative effect sizes indicate that the control group had smaller declines in both self-report and performance-based physical function compared with the mHealth-based and paper-based groups.

## Discussion

Findings of this study indicate that self-management interventions consisting of one in-person visit and 3 phone calls may be efficacious in promoting physical activity among adults with different musculoskeletal and neurological conditions. We found few differences between the mHealth-based and paper-based self-management interventions. Thus, further research is needed to determine the best ways to use mHealth apps in self-management interventions. Hypothesis #1 was partially supported by the results, while hypotheses #2 and #3 were not supported by the results. In regard to hypothesis #1 for physical activity levels, we found that both the mHealth-based and paper-based groups had moderate to large effect size increases in exercise and leisure-time physical activity compared with the control group. In addition, the mHealth-based group had a small to moderate effect size increases in planned exercise and leisure-time physical activity compared with the paper-based group. In regard to hypothesis #2 for psychosocial factors, we found that there were no significant differences between the 3 groups, but both the mHealth-based and paper-based groups had small to moderate effect size improvements in psychosocial factors compared with the contact-control group. In regard to hypothesis #3 for physical function, we found that there were no significant differences between the 3 groups, and there were small and nonsignificant declines in physical function.

### Physical Activity

The composite score of PADs-revised indicated that participants in the mHealth-based and paper-based groups increased their engagement in physical activity, whereas participants in the contact-control group decreased their engagement in physical activity. However, participants in the contact-control group reported increased engagement in the general physical activity subscale of the PADs-revised, which helps explain the negative effect sizes for general physical activity levels among participants in the mHealth-based and paper-based groups. It will be important that future clinical trials measure different types of physical activities to determine whether participants are increasing their overall physical activity levels or substituting one type of physical activity with another type of physical activity.

Several randomized controlled trials of physical activity interventions have shown that research participants in control groups change their physical activity habits. Waters et al [46] found in a systematic literature review that screening to exclude physically active participants and include participants with chronic conditions was related to meaningful control group improvements in physical activity. Changes in physical activity habits among participants in the control intervention group may also be due to the completion of questionnaires on physical activity, information on the benefits of physical activity, and the eagerness of participants to make changes in their physical activity levels (ie, selection bias). Thus, future clinical trials of physical activity interventions in adults with musculoskeletal and neurological conditions will need to ensure sample sizes are of a sufficient size to overcome the possibility that participants in the control groups may increase physical activity levels.

Participants were significantly more likely to track physical activity behaviors using the paper diary compared with the mHealth app. However, using the paper diary did not result in significant increases in physical activity or better health outcomes. In fact, effect sizes indicate that participants in the mHealth-based group had moderate increases in exercise and leisure-time physical activity compared with the paper-based group. These results may indicate that the benefits of augmenting mHealth apps in a self-management intervention may not entirely be due to the functionalities of tracking physical activity behaviors and receiving feedback. As described in the introduction, augmenting mHealth apps in self-management interventions may also facilitate the tailoring of information that may help promote behavior change. Providing participants with the tablet at the beginning of the intervention may also have fostered a stronger sense of commitment to the study, which facilitated engagement in physical activity. Given the cost associated with mHealth technology, there is a need to conduct future qualitative and quantitative research to identify the best ways of augmenting mHealth apps in self-management interventions.

### Psychosocial Factors

To understand why there may be benefits of augmenting mHealth apps in self-management interventions consisting of in-person visits and phone calls, additional measures may need to be incorporated beyond the common psychosocial factors that were measured in this study (ie, self-efficacy, self-regulation, and social support). There were small and nonsignificant differences between the mHealth-based and paper-based groups on psychosocial factors. Thus, this result provides limited insight into why there may have been differences between the mHealth-based and paper-based groups in terms of promoting planned exercise and leisure-time physical activity. Nonetheless, these psychosocial factors may provide insight on how both the mHealth-based and paper-based self-management interventions could be improved in future studies. For example, both the mHealth-based and paper-based self-management interventions had a small effect on social support, which means there may be opportunities to incorporate additional behavior change strategies to promote physical activity. Incorporating social networking functionality of mHealth apps into a self-management intervention may be a strategy to promote social support for engaging in physical activity.

### Physical Function

Participants in the mHealth-based and paper-based groups did not have improvements in physical function. Several factors may explain this finding. First, it may be that 6 weeks is simply too short a time span to detect benefits of promoting physical activity. Second, we failed to recruit participants with more severe limitations in physical function, which may have contributed to ceiling effects. Third, adults with different musculoskeletal and neurological conditions engaging in different types of physical activity programs created heterogeneity in responses, which may have led to small average changes. Inclusion of adults with different conditions and accommodating preferences for engaging in physical activity programs is more consistent with patient-centered clinical care [47]. Thus, future studies with a larger sample size and long-term follow-up are needed to examine heterogeneity in responses to different types of physical activity programs that are prescribed to participants with different musculoskeletal and neurological conditions.

### Relevance to Existing Research

Most literature reviews of mHealth apps to promote healthy behaviors generally conclude that there is a need for more rigorous research using randomized controlled trial designs [16,18-20,48-53]. Researchers who have restricted reviews to a particular type of chronic condition have generally identified promising studies on the usability of mobile apps, but there is little evidence on the apps’ effectiveness to promote healthy behaviors [48-50,54,55]. Researchers who have conducted reviews with a broader focus across populations with disabling conditions indicate that mHealth apps have small to moderate effect sizes in terms of promoting healthy behaviors [16,51,52,56]. Difference in effect sizes may depend upon the type of control group used in the clinical trial and the heterogeneity in the functional capacity of participants. Our results are consistent with these meta-analyses and help advance existing research by demonstrating the feasibility of conducting clinical trials of mHealth technology across a population of adults with different disabling conditions who are inexperienced in using mobile devices to track behaviors.

Our study is one of the first studies to compare mHealth apps with paper diaries in promoting physical activity among adults with disabling conditions. There has been some research on weight loss in the general population comparing mHealth apps with paper diaries [57-60]. These studies have found that mHealth apps can result in better tracking and health behavior outcomes compared with a paper diary. A possible explanation for these inconsistencies with our results is that we had fewer interactions with our participants using the mHealth apps. For example, Tuner-McGrievy et al [57] sent podcasts twice a week to participants and asked participants to report their frequency of tracking physical activity weekly. They found that participants randomized to the mHealth group showed improved tracking of physical activity behavior compared with participants that did not use the app. Thus, having frequent interactions with participants may help to remind and motivate participants to track their behaviors using mHealth apps. Research is needed to identify the fewest number of interactions needed to motivate consistent use of mHealth apps.

There is a growing research literature on using mHealth apps on a mobile phone to promote physical activity in the general population. Several studies have shown that real-time tracking with a mobile phone and providing personalized feedback can promote physical activity in adults with cardiovascular risk factors [61-63]. Lobelo et al outlined a model to integrate mobile phones and wearables into health care settings [64]. We decided to use a tablet instead of a mobile phone because we thought that the larger screen size would reduce usability barriers. However, the disadvantage of using a tablet is that it may be impractical to use real-time tracking features to monitor physical activity levels. Thus, both the feasibility and accuracy of using a mobile phone to promote and monitor physical activity levels in adults with disabling conditions need to be explored in future research studies.

### Limitations

A limitation of our study was that the full potential of mHealth apps was not utilized; features such as social networking, tailored text messages, motion sensors, and global positioning systems were not used. Some of these functionalities may cost extra if used in commercially available apps or may not be relevant to adults with disabling conditions, and may increase the risk to patient privacy. Thus, several researchers have developed their own mobile apps, with moderate success in promoting self-management behaviors and improving health outcomes [21,65-70]. A potential limitation to this approach is expansion of mHealth apps without the creation of knowledge about the best ways to use mHealth apps in self-management interventions. Circumventing this potential limitation will require collaborations between clinicians, computer scientists, and researchers who conduct comparative effectiveness research to identify the best ways to use mHealth apps in self-management interventions [71].

Other limitations to this study include the small sample size, increased risk of type I error, lack of generalizability, and self-report bias. The small sample size may have resulted in overestimation of effect sizes and lowered probability of reproducing the results. Because this was considered a pilot study, we conducted post hoc analyses without controlling for family-wise error, which increased the likelihood of making a type I error. Results of this study may not be generalizable to adults with more severe physical limitations or adults who are unable to walk. Use of self-report measures of physical activity may have resulted in participants exaggerating engagement in physical activity at the posttest and may have further exaggerated effect sizes. Future studies should measure physical activity levels using an accelerometer. Furthermore, there is a need to examine the validity and reliability of Goal Setting for Exercise Scale, Social Support and Exercise Survey, and Exercise Confidence Survey across a population segment with different musculoskeletal or neurological conditions. In this paper, we did not report on nutritional outcomes or a qualitative process evaluation (eg, participants’ subjective experience in the interventions). We intend to publish these results in the future.

### Conclusions

Comparing mHealth-based to paper-based self-management interventions may provide insight into the added value of using mHealth apps in self-management interventions, as well as the ways in which mHealth apps can be improved. We found that both the mHealth-based and paper-based self-management interventions consisting of one in-person visit plus 3 phone calls may be efficacious in promoting physical activity. However, the mHealth-based self-management intervention was not significantly different from the paper-based self-management intervention. There is a need to conduct further research using a larger sample size, incorporating objective measures of physical activity, and having a long-term follow-up period in order to validate the results of this study and address its limitations.

## Abbreviations

GAS: goal attainment scaling
MANOVA: multivariate analysis of variance
PADS-revised: Physical Activity and Disability Survey–revised
PROMIS: Patient Reported Outcomes Measurement Information System

## Appendix

Multimedia Appendix 1 CONSORT‐EHEALTH checklist (V 1.6.1).
